# Neurological and Immunological Insights Into Post-COVID-19 Syndrome: A Single-Center Retrospective Study

**DOI:** 10.7759/cureus.88098

**Published:** 2025-07-16

**Authors:** Lara Diem, Simona Daepp, Vincenzo P Introcaso, Helly Hammer, Christoph Friedli, Nicole Kamber, Andrew Chan, Anke Salmen, Robert Hoepner

**Affiliations:** 1 Neurology, Lucerne Cantonal Hospital, Lucerne, CHE; 2 Neurology, University Hospital Bern and University of Bern, Bern, CHE; 3 Neurology, University Hospital Bern, Bern, CHE; 4 Neurology, Waikato Hospital, Hamilton, NZL; 5 Neurology, Bürgerspital Solothurn, Solothurn, CHE; 6 Neurology, St. Josef Hospital, Ruhr University Bochum, Bochum, DEU

**Keywords:** biomarkers, coronavirus, follow-up, long-term symptoms, neuropsychiatric symptoms, postinfectious, sars-cov-2, viral infection

## Abstract

Introduction

Following the coronavirus disease 2019 (COVID-19) pandemic, caused by severe acute respiratory syndrome coronavirus 2 (SARS-CoV-2), post-COVID-19 syndrome (PCS) has emerged as a major health concern, affecting approximately 3.0-11.7% of infected individuals. While neuropsychiatric symptoms such as fatigue, pain, and sleep disturbances dominate the clinical picture, recent evidence suggests a pivotal role for immune dysregulation in the pathophysiology of PCS. This study aimed to analyze the clinical course and laboratory features of patients with PCS.

Methods

In this retrospective single-center study, 74 patients with PCS were evaluated between November 2020 and June 2022 at the Department of Neurology, University Hospital Bern. Clinical assessments included standardized questionnaires [Fatigue Severity Scale (FSS), Fatigue Scale of Motor and Cognitive Function (FSMC), Beck Depression Index II (BDI-II), Epworth Sleepiness Scale (ESS)] and laboratory investigations at two timepoints. Particular focus was placed on immune-related biomarkers, including ferritin and antinuclear antibodies (ANA).

Results

Fatigue, sleep disturbances, and pain were the most frequently reported symptoms. At follow-up (a mean duration of 14.4 weeks after the first consultation), both symptom burden and severity scores decreased significantly. Nevertheless, 82.4% of patients continued to report fatigue. Elevated ferritin levels were found in 78.1% of tested patients (mean: 306.9 µg/l), and ANA titres ≥1:160 were observed in 40% of patients without known autoimmune disease. At follow-up, 80% of patients with initially elevated ferritin and 68% with elevated ANA remained above normal thresholds, suggesting ongoing immune activation.

Conclusions

While symptom burden declined over time, most PCS patients continued to experience clinically relevant symptoms. The persistence of elevated ferritin and ANA levels underscores possible immune dysregulation and highlights the potential of these biomarkers in characterizing PCS. These findings underline the need for further studies investigating their diagnostic and therapeutic relevance.

## Introduction

In the wake of the coronavirus disease 2019 (COVID-19) pandemic, caused by severe acute respiratory syndrome coronavirus 2 (SARS-CoV-2), the emergence of post-COVID-19 syndrome (PCS) has raised significant concerns, and it affects 3.0-11.7% of patients after initial infection [1]. Defined as “the continuation or development of new symptoms three months after the initial SARS-CoV-2 infection, with these symptoms lasting for at least two months with no other explanation” by the WHO [2], PCS is associated with more than 200 different symptoms. The most frequently mentioned symptoms in the current literature are sleep disturbances, fatigue, and pain, usually closely followed by cognitive, psychiatric (especially depressive mood), and respiratory symptoms (dyspnea and cough) [1,3,4].

The pathogenesis of PCS remains a subject of intense investigation. While initial hypotheses suggested persistent organ damage or viral reservoirs, recent studies highlight immune dysregulation as a primary mechanism​​ [5]. Key findings include the persistent activation of innate and adaptive immune responses, elevated levels of proinflammatory cytokines [e.g., interleukin-1 beta (IL-1β), IL-6, and tumor necrosis factor alpha (TNF-α)], and evidence of endothelial dysfunction and microclot formation​​ [6]. Additionally, SARS-CoV-2 may promote hyperferritinemia via its intrinsic hepcidin-mimetic effect, exacerbating systemic inflammation​ [7].

Emerging evidence strongly implicates autoimmunity in the PCS. Circulating antinuclear antibodies (ANAs) and other autoantibodies, including anticardiolipin antibodies, have been detected in COVID-19 survivors up to 12 months post-infection, with a higher prevalence in those experiencing persistent symptoms such as fatigue and dyspnea​​​ [6, 8, 9]. Persistent ANA positivity has been linked to chronic inflammation, as evidenced by elevated TNF-α and C-reactive protein (CRP) levels, and is considered a marker for ongoing immune activation [8]. Furthermore, the persistence of immunoglobulin G (IgG) anticardiolipin antibodies has been associated with post-COVID-19 complications, including microvascular and neurological impairments, pointing to their role as potential biomarkers for PCS​ [9].

With respect to the disease course, studies indicate that more than 70% of PCS patients experience persistent symptoms one year after infection, with this proportion decreasing to 10-63% after two years​​ [10-13]. The syndrome has severe implications for patients’ quality of life and economic productivity. For example, a Danish study reported that 1.5% of patients did not return to work six months post-infection, whereas other studies have shown significant impairments in social and work life​ [14]. In another study, social life was affected in 15% of 323 patients followed up at eight months, whereas work capacity was impaired in 8% of patients at the same time [15].

This study aimed to longitudinally investigate the clinical course and laboratory characteristics of patients with PCS. Specifically, we sought to (1) describe the evolution of core PCS symptoms-namely fatigue, daytime sleepiness, and depression-over time, (2) determine whether symptom burden decreases during the natural course of the condition, and (3) explore the persistence and potential relevance of laboratory alterations such as elevated ferritin and ANAs in the months following acute SARS-CoV-2 infection. By focusing on both symptom progression and immune-related laboratory findings, this study aimed to contribute to a better understanding of the pathophysiology and recovery patterns in PCS and to identify potential biomarkers that may assist in monitoring or stratifying affected individuals.

## Materials and methods

A total of 295 patients seen in our clinic met the criteria for the diagnosis of PCS, of whom 182 patients underwent clinical follow-up (also referred to as a second consultation). We included only those patients with clinical follow-up data and who completed questionnaires during both consultations (n=74) in this retrospective single-center study. All of them agreed to participate in the prospective neuroimmunological registry. The included patients were seen between 11/2020 and 06/2022 at our consultation at the Department of Neurology, University Hospital Bern in Switzerland. Parts of the consultation included a detailed history regarding the current symptoms and the symptoms of the acute infection, as well as a personal anamnesis, including previous health issues, medication, and psychosocial situation.

Furthermore, questionnaires on mood [Beck Depression Index II (BDI-II)], daytime sleepiness [Epworth Sleepiness Scale (ESS)], and fatigue [Fatigue Severity Scale (FSS) and Fatigue Scale of Motor and Cognitive Function (FSMC)] were completed. Fatigue was defined as an FSMC score ≥43 points or an FSS score ≥4 points. For the FSMC score, ≥43 points was considered mild, ≥53 points was considered moderate, and ≥63 points was considered severe fatigue [16,17]. Scores lower than 43 points were considered "no fatigue". Scores of 0-8 points on the BDI-II were considered "no depression", scores of 9-13 points were considered minimal, scores of 14-19 points were considered mild, scores of 20-28 points were considered moderate, and scores of 29-63 points were considered severe depression [18]. Daytime sleepiness was defined as an ESS score ≥11 points [19]. To assess changes in the questionnaires (FSMC, BDI-II, ESS), we calculated category changes as per Hubacher et al. [20]. For example, a patient who was in the "severe fatigue" category at the first consultation and in the "moderate fatigue" category at follow-up was shifted to a less fatigued category. A clinically minimal improvement in the FSS score was defined as a decrease of at least 0.5 points [21].

All necessary permissions for the use of the BDI-II, ESS, FSS, and FSMC questionnaires were obtained. The hospital holds institutional licenses where required, and the use of all instruments was conducted in compliance with applicable copyright and licensing regulations (tables in the Appendices). Anamnesis and scoring were followed by a physical examination and a detailed laboratory evaluation (see table in the Appendices). In our study, we focused on acute phase proteins (CRP, ferritin) and autoantibodies (anti-cardiolipin/-b2-glycoprotein-antibodies, ANAs). Follow-up laboratory analyses were performed only in patients who had abnormal values at baseline. This approach may introduce selection bias and limit the comparability and reproducibility of results across different populations and settings.

Quantitative variables are described using the mean and 95% confidence interval (95% CI) and were compared with the Wilcoxon signed rank test. Qualitative variables are presented as absolute numbers and frequencies and were compared with the chi-square test and the McNemar test. Adjustment for multiple testing was performed via the Bonferroni procedure for each domain. The statistical package used was IBM SPSS Statistics version 29 (IBM Corp., Armonk, NY).

Ethics Declaration: This study was conducted in accordance with the principles of the Declaration of Helsinki. Ethical approval was obtained from the IRB (no: 2017-01369).

## Results

Characteristics of the cohort

The majority of patients in the study were female, accounting for 61 out of 74 (82.4%) of the patients. The mean age was 44.0 years (95% CI: 41.1-47.0, n=74). The initial consultation occurred at an average of 35.0 weeks (95% CI: 30.8-39.2, n=74), approximately eight months following the acute phase of COVID-19, with most patients being referred externally for evaluation. Follow-up occurred on average 14.4 weeks after the first consultation (95% CI: 12.5-16.3, n=74), which corresponds to approximately 12 months after the initial SARS-CoV-2 infection. COVID-19 infection was confirmed in 95.9% (71/74) of the patients by a positive PCR test and in 3/74 (4.1%) of the patients by serological detection of anti-nucleocapsid IgG. The infection was severe/critical, with hospitalization in 8/74 (10.8%) patients and the need for intubation in 1/74 (1.4%) patients. Comorbidities were present in 19 of 74 patients (25.7%). The most common comorbidities were bronchial asthma (7/74 (9.5%) and depression (5/74 (6.8%)), followed by diabetes mellitus, hypothyroidism, and hypertension [each 2/74 (2.7%)]. A summary of the cohort's characteristics is presented in Table 1.

**Table 1:** Characteristics of all patients with post-COVID-19 syndrome at the first consultation ^*^Education over 13 years corresponds to compulsory schooling (nine years) and job training (three years) in Switzerland. **Ferritin analysis without patients with iron deficiency. Iron deficiency was defined as ferritin <50 µg/l and transferrin saturation <16% Statistics: quantitative variables are described using the mean and 95% confidence interval (95% CI); qualitative variables are presented as absolute numbers and frequencies CI: confidence interval; PCR: polymerase chain reaction; antibody test: antibody test against anti-nucleocapsid-IgG; recurrent fever: repeatedly measured temperature over 38.0 °C by the patients themselves; FSS: Fatigue Severity Score; FSMC: Fatigue Scala for Motor and Cognitive Function; ESS: Epworth Sleepiness Scale; BDI-II: Beck Depression Index II; CRP: C-reactive protein, reference <5 mg/l; ferritin: reference - men: 50–140 µg/l, women: 50–156 µg/l; ANA: antinuclear antibodies, normal: <1:80, borderline elevated: 1:80–1:160, moderately elevated: 1:160–1:640, strongly elevated: ≥1:1280; anti-cardiolipin- and anti-b2-glycoprotein-antibodies: reference: <20 chemiluminescent units

Variable	Values		
Population characteristics	N=74		
Age, years, mean (95% CI)	44.0 (41.1-47.0)		
Female sex, n (%)	61 (82.4)		
Education ≥13 years*, n (%)	49 (66.2)		
Time between onset of acute infection and first consultation, weeks, mean (95% CI)	35.0 (30.8-39.2)		
Time between first consultation and follow-up, weeks, mean (95% CI)	14.4 (12.5-16.3)		
Time between infection and follow-up, months, mean (95% CI)	10.8 (9.8-11.3)		
Estimated duration of acute COVID-19, days, mean (95% CI)	16.4 (14.6-18.2)		
Comorbidities, n (%)	19 (25.7)		
Hypertension	2 (2.7)		
Diabetes mellitus	2 (2.7)		
Depression	5 (6.8)		
Hypothyroidism	2 (2.7)		
Bronchial asthma	7 (9.5)		
Cancer, n (%)	1 (1.4)		
Acute COVID-19 infection characteristics	N=74		
Symptoms of acute COVID-19, n (%)			
Headache	43 (58.1)		
Fever	49 (66.2)		
Anosmia	48 (64.9)		
Dyspnea	36 (48.6)		
Cough	50 (67.6)		
Cold	42 (56.8)		
Pain	50 (67.6)		
Gastrointestinal symptoms	17 (23.0)		
Fatigue	56 (75.7)		
Sleep disturbance (difficulty falling asleep and/or sleep maintenance insomnia), n (%)	34 (45.9)		
Hospitalization, n (%)	8 (10.8)		
Intubation, n (%)	1 (1.4)		
Positive PCR/antibody Test, n (%)	74 (100)		
Positive PCR test	71 (95.9)		
Positive antibodies against anti-nucleocapsid-IgG	3 (4.1)		
Post-COVID-19 syndrome symptoms (reported by patients, subjective), n (%)	N=74		
Fatigue	74 (100)		
Headache	33 (44.6)		
Muscle/joint pain	28 (37.8)		
Neuropathic pain	11 (14.9)		
Chest pain	11 (14.9)		
Sleep disturbance	50 (67.6)		
Dyspnea	31 (41.9)		
Cough	4 (5.4)		
Depressive mood, feeling of anxiety	20 (27.0)		
Dizziness	25 (33.8)		
Anosmia/ageusia	2 (2.7)		
Autonomic dysfunction	26/74 (35.1)		
Gastrointestinal symptoms, n (%)	0 (0)		
Tinnitus, n (%)	1 (1.4)		
Total number of symptoms at first consultation, mean (95% CI)	4.4 (3.9-4.9)		
Questionnaire scores, mean (95% CI)	N=74		
FSS	5.7 (5.4-5.9)		
FSMC total	78.5 (75.5-81.4)		
FSMC cognition	39.7 (37.9-41.5)		
FSMC motor	38.7 (37.2-40.3)		
ESS	10.0 (8.9-11.0)		
ESS ≥11 points, n (%)	33 (44.6)		
BDI-II, mean (95% CI)	18.0 (15.8-20.2)		
BDI-II ≥14 points (mild depression), n (%)	46 (62.2)		
Laboratory analysis			
Acute phase proteins	N=32		
Ferritin^**^, mean (95% CI)	306.9 (240.0-373.8)		
CRP, mean (95% CI)	6.1 (-0.3-12.5)		
Elevated ferritin, n (%)	25 (78.1)		
Elevated CRP, n (%)	1 (3.1)		
ANA, n (%)	N=50		
Weakly elevated ANA	29 (58.0)		
Moderately elevated ANA	18 (36.0)		
Strongly elevated ANA	2 (4.0)		
Anti-cardiolipin or anti-b2-glycoprotein-antibodies	N=14		
Anti-cardiolipin or anti-b2-glycoprotein-antibodies elevated, n (%)	8 (57.1)		

Clinical phenotype of post-COVID-19 syndrome (PCS)

The three most common symptoms in our cohort were fatigue, pain, and sleep-wake disturbances. All patients (74/74, 100%) reported experiencing fatigue. The average severity of fatigue, as measured by FSS, was 5.7 (95% CI: 5.4-5.9, n=74), and the mean FSMC score was 78.5 points (95% CI: 75.5-81.4, n=74). Compared with motor fatigue, cognitive fatigue was slightly more pronounced (FSMC cognition: mean 39.7, 95% CI: 37.9-41.5, n=74) (FSMC motor: mean: 38.7, 95% CI 37.2-40.3, n=74). Additionally, 50/74 (67.6%) of the participants reported experiencing sleep disturbances, including difficulty falling asleep and/or maintaining sleep. The mean reported daytime sleepiness severity on the ESS was 10.0 points (95% CI: 8.9-11.0, n=74), and 33/74 (44.6%) of the respondents reported excessive daytime sleepiness (defined by ESS score ≥11 points).

The most frequently reported type of pain was headache (33/74, 44.6%), followed by muscle and joint pain (28/74, 37.8%). Psychiatric symptoms were also common. In fact, 20/74 (27.0%) of the patients reported subjective psychiatric complaints, such as anxiety and depressed mood. The mean BDI-II score was 18.0 (95% CI 15.8-20.2, n=74), and 46/74 (62.2%) of the patients had a score corresponding to at least mild depression (BDI ≥14 points). At the first consultation, patients had an average of 4.4 symptoms (95% CI 3.9-4.9, n=74).

Acute-phase proteins and autoantibodies

Ferritin levels were analyzed during both the first and second consultations in 32 out of the 74 patients. The mean ferritin concentration at first consultation was 306.9 µg/l (95% CI: 240.0-373.8 µg/l, n=32; reference: men: 80-140 µg/l, women: 50-156 µg/l)[22]. In total, 25/32 (78.1%) patients had elevated ferritin levels. Most of the patients had normal CRP levels [mean: (95% CI) 6.1 (-0.3-12.5), n=32] (reference: <5 mg/l)[23]. Elevated CRP values were detected in only 1/32 (3.1%) patients. Elevated ANA levels (≥1:160) were present in 20/50 (40%) of patients without known or new evidence of autoimmune disease. More than half of the patients had elevated anti-cardiolipin/anti-glycoprotein antibodies without having known or new antiphospholipid syndrome or without fulfilling the Sapporo criteria for the laboratory diagnosis of antiphospholipid syndrome.

Second consultation: symptoms and scores

At the second consultation, patients presented with a mean of 3.2 symptoms (95% CI: 2.9-3.8; n=74), which was significantly lower than that at the first consultation [mean (95% CI): 4.4 (3.9-4.9); n=74; p<0.001]. Fatigue was still present in 61/74 (82.4%) patients at the second consultation. However, we observed a relevant decrease in prevalence (first consultation: 74/74 (100%), p<0.001). Sleep disturbance decreased significantly between the first consultation and follow-up [50/74 (67.6%) vs. 40/74 (54.1%), p<0.001]. Additionally, autonomic dysfunction [26/74 (35.1%) vs. 14/74 (18.9%), p=0.004] significantly decreased between the first consultation and follow-up (Table 2).

**Table 2:** Patients with complete reported symptoms, scores and laboratory analysis: first vs. second consultation ^*^Ferritin analysis without patients with iron deficiency. Iron deficiency was defined as ferritin <50 µg/l and transferrin saturation <16% Statistics: quantitative variables are described using the mean and 95% CI, and they were compared with the Wilcoxon signed rank test (z-value). Qualitative variables are presented as absolute numbers and frequencies and were compared with the chi-square test and McNemar test (both chi-square values). Adjustment for multiple testing was performed via the Bonferroni procedure for each domain: post-COVID-19 syndrome symptoms (reported by patients, subjective): p<0.001; questionnaire scores: p=0.01; laboratory analysis: p=0.01 CI: confidence interval; PCR: polymerase chain reaction; antibody test: antibody test against anti-nucleocapsid-IgG; recurrent fever: repeatedly measured temperature over 38.0 °C by the patients themselves; FSS: Fatigue Severity Score; FSMC: Fatigue Scala for Motor and Cognitive Function; ESS: Epworth Sleepiness Scale; BDI-II: Beck Depression Index II; CRP: C-reactive protein, reference <5 mg/l; ferritin: reference - men: 50–140 µg/l, women: 50–156 µg/l; ANA: antinuclear antibodies, normal: <1:80, borderline elevated: 1:80–1:160, moderately elevated: 1:160–1:640, strongly elevated: ≥1:1280; anti-cardiolipin- and anti-b2-glycoprotein-antibodies: reference: <20 chemiluminescent units

Variable	First consultation	Second consultation	Chi-square value/z-value	P-value
Post-COVID-19 syndrome symptoms (reported by patients, subjective), n (%)	N=74	N=74		
Fatigue	74 (100)	61 (82.4)	13.0	<0.001
Headache	33 (44.6)	25 (33.8)	1.79	0.180
Muscle/joint pain	28 (37.8)	22 (29.7)	3.17	0.070
Neuropathic pain	11 (14.9)	11 (14.9)	0	1.0
Chest pain	11 (14.9)	6 (8.1)	3.20	0.063
Sleep disturbance	50 (67.6)	40 (54.1)	8.10	<0.001
Dyspnea	31 (41.9)	20 (27.0)	9.09	0.021
Cough	4 (5.4)	2 (2.7)	0.50	0.500
Depressive mood, feeling of anxiety	20 (27.0)	16 (21.6)	0.48	0.572
Dizziness	25 (33.8)	22 (29.7)	1.33	0.267
Anosmia/ageusia	2 (2.7)	2 (2.7)	0	1.0
Autonomic dysfunction	26 (35.1)	14 (18.9)	10.08	0.004
Gastrointestinal symptoms	0 (0)	0 (0)	0	1.0
Tinnitus	1 (1.4)	1 (1.4)	0	1.0
Total number of symptoms at second consultation, mean (95% CI)	4.4 (3.9-4.9)	3.2 (2.9-3.8)	-4.06	<0.001
Questionnaire scores, mean (95% CI)	N=74	N=74		
FSS	5.7 (5.4-5.9)	5.2 (4.9-5.5)	-2.83	<0.001
FSMC total	78.5 (75.5-81.4)	70.9 (67.0-74.9)	-2.82	<0.001
FSMC cognition	39.7 (37.9-41.5)	36.0 (33.8-38.1)	-2.59	0.003
FSMC motor	38.7 (37.2-40.3)	34.9 (32.9-36.9)	-2.77	<0.001
ESS	10.0 (8.9-11.0)	8.3 (7.3-9.3)	-2.88	<0.001
BDI-II	18.0 (15.8-20.2)	13.9 (12.2-15.5)	-4.49	<0.001
Laboratory analysis				
Acute phase proteins	N=32	N=32		
Ferritin^*^, mean (95% CI)	306.9 (240.0-373.8)	218.2 (164.7-271.8)	-4.50	<0.001
CRP, mean (95% CI)	6.1 (-0.3-12.5)	3.3 (2.6-4.1)	-1.34	0.128
ANA level, n (%)	N=50	N=50		
Weakly elevated ANA	29 (58.0)	39 (78.0)	6.13	0.022
Moderately elevated ANA	18 (36.0)	10 (20.0)	2.29	0.113
Strongly elevated ANA	2 (4.0)	1 (2.00)	0.33	0.56
Anti-cardiolipin or anti-b2-glycoprotein-antibodies	N=14	N=14		
Anti-cardiolipin or anti-b2-glycoprotein-antibodies elevated, n (%)	8 (57.1)	4 (28.6)	1.33	0.25

Second consultation: change in scores

Fatigue Scores

In both fatigue questionnaires (FSS and FSMC), there was a decrease in the scores at follow-up (see Table 2). The mean total FSS score at follow-up was 5.2 (95% CI: 4.9-5.5, n=74), which was still severe fatigue; however, it was lower than that at the first consultation [mean (95% CI): 5.7 (5.4-5.9), n=74, p<0.001]. A clinically minimal improvement in the FSS score (defined by a decrease in the FSS score of at least 0.5 points [21]) was found in 28/74 patients (37.8%) (Table 3).

**Table 3:** Differences in questionnaire scores between the first and second consultations ^*^A decrease of at least 0.5 points in the FSS score was defined as clinically minimal improvement PCS: post-COVID-19 syndrome; FSS: Fatigue Severity Score; FSMC: Fatigue Scale for Motor and Cognitive Function (fatigue: >43 points, ≥43 points: mild fatigue, ≥53 points: moderate fatigue, ≥63 points: severe fatigue); ESS: Epworth Sleepiness Scale (daytime sleepiness: >11 points); BDI-II: Beck Depression Index II (0–8 points: no depression, 9–13 points: minimal depression, 14–19 points: mild depression, 20–28 points: moderate depression, 29–63 points: severe depression)

Change in scores	PCS patients
FSS decrease 0.5 points^*^, n (%)	28/74 (37.8)
FSMC never-fatigued patients, n (%)	5/74 (6.8)
FSMC no category change in follow-up, n (%)	53/74 (71.6)
FSMC category-improvement in follow-up total, n (%)	18/74 (24.3)
FSMC improvement 1 category, n (%)	8/74 (10.8)
FSMC improvement 2 categories, n (%)	8/74 (10.8)
FSMC improvement 3 categories, n (%)	2/74 (2.7)
FSMC category-deterioration in follow-up total, n (%)	3/74 (4.1)
FSMC deterioration 1 category, n (%)	3/74 (4.1)
FSMC deterioration 2 categories, n (%)	0/74 (0)
FSMC newly fatigued patients in follow-up, n (%)	0/74 (0)
ESS persistent daytime sleepiness (patients with no daytime sleepiness ever excluded), n (%)	23/74 (31.2)
ESS no more daytime sleepiness in follow-up, n (%)	17/74 (23.0)
ESS new onset of daytime sleepiness in follow-up, n (%)	4/74 (5.4)
BDI-II: never depressed, n (%)	28/74 (37.8)
BDI-II: same category in follow-up, n (%)	36/74 (48.6)
BDI-II: less depressed category in follow-up, n (%)	31/74 (41.9)
By one category, n (%)	21/74 (28.4)
By two categories, n (%)	7/74 (9.4)
By three categories, n (%)	3/74 (4.1)
BDI-II: more depressed category in follow-up, n (%)	7/74 (9.4)
By one category, n (%)	5/74 (6.8)
By two categories, n (%)	2/74 (2.7)

**Figure 1:**
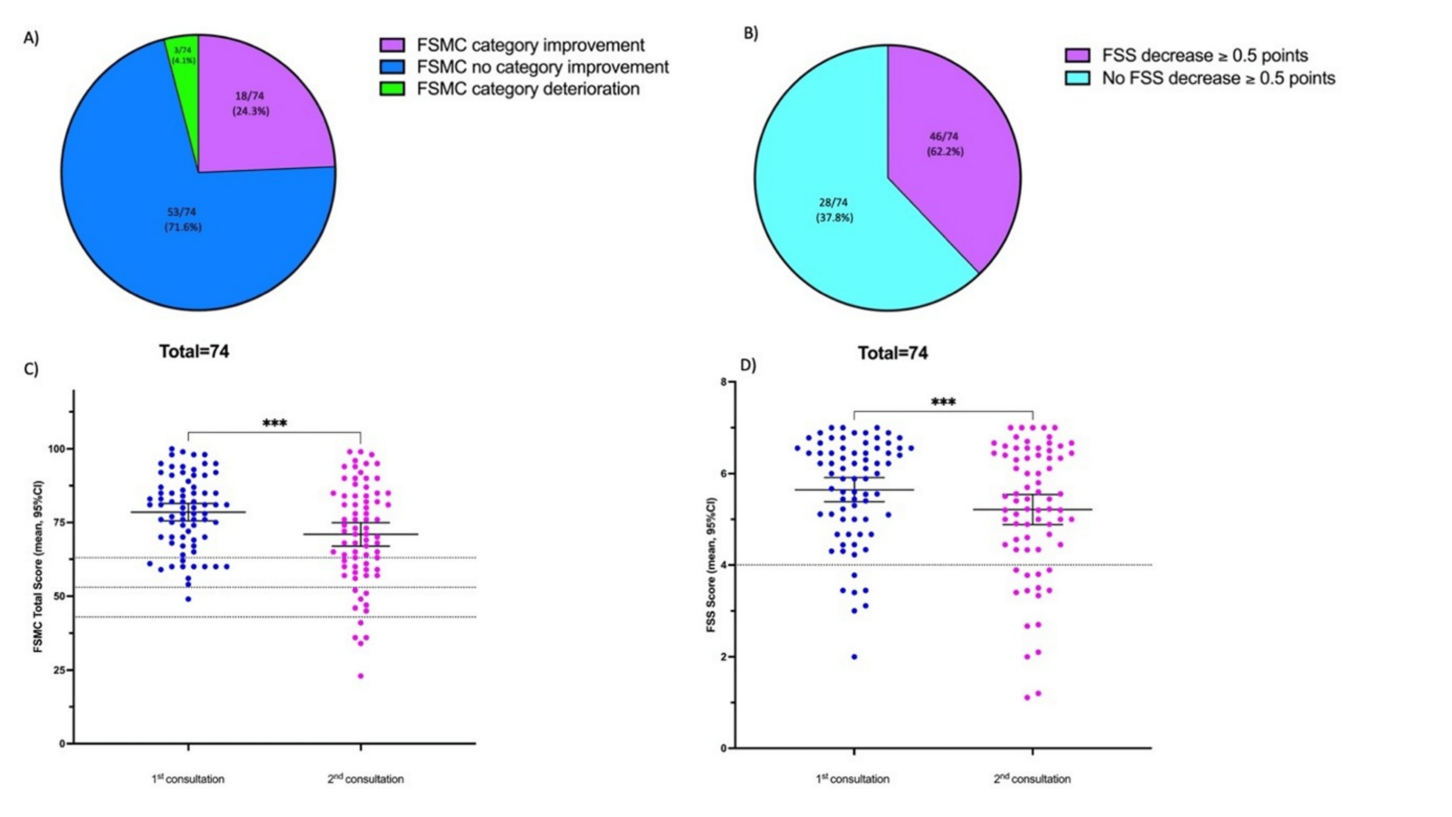
Evolution of fatigue scores between the first and second consultation (FSMC and FSS) A, C: changes in the severity of FSMC at the second consultation. B, D: changes in the severity of FSS at the second consultation FSMC: Fatigue Scale of Motor and Cognitive Function; FSS: Fatigue Severity Scale

The mean total FSMC score at follow-up was 70.9 (95% CI: 67.0-74.9, n=74), which still corresponded to severe fatigue. Most of the patients remained in the same severity category (53/74, 71.6%). A total of 8/74 (10.8%) patients became less fatigued by one category, 8/74 (10.8%) became less fatigued by two categories, and 2/74 (2.7%) became less fatigued by three categories. In summary, the FSMC score of 18/74 (24.3%) of the patients changed to a less fatigued category at follow-up (Table 3, Figure 1). On the other hand, 3/74 (4.1%) patients became fatigued at follow-up. No patient with a new onset of fatigue in FSMC was found (Table 3).

Daytime Sleepiness and Depression Scores

Approximately 1/3 of the patients (23/74, 31.2%) experienced persistent daytime sleepiness at follow-up. A total of 17/74 (23.0%) patients showed no more daytime sleepiness during the follow-up ESS. At follow-up, 16/74 (21.6%) of patients reported subjective psychiatric symptoms, such as depressive mood and/or anxiety. BDI-II, which had an initial mean score of 18.0 points at the first consultation, showed significantly lower scores at the second consultation, with a mean score of 13.9 (95% CI: 12.2-15.5, n=74) (Table 3). Approximately half of the patients (36/74, 48.6%) did not show any change in the category of severity of depression, 31/74 (41.9%) moved to a less depressive category, and 7/74 (9.4%) reported a higher score on the BDI-II (Table 3).

Second consultation: laboratory analysis

Laboratory analysis was only repeated in cases of abnormalities in the first analysis. Among the 25 patients with initially elevated ferritin levels, 20/25 (80%) continued to have ferritin levels within the pathological range (above 150 µg/L) at follow-up. On average, ferritin levels decreased by 88.7 µg/L (95% CI: 48.4-128.9, n=32) from the first consultation to follow-up. Specifically, the mean ferritin level at the first consultation was 306.9 µg/L (95% CI: 240.0-373.8, n=32), whereas it was 218.2 µg/L (95% CI: 164.7-271.8, n=32) at the follow-up, reflecting a statistically significant reduction (p<0.001, Tables 2, 4). There was no correlation between improvement in FSS and a decrease in ferritin (p=0.918), but patients with improvement in FSS (more than 0.5 points) initially had higher ferritin levels than those with no improvement [with improvement: mean ferritin (95% CI): 257.7 (155.8-359.6), n=28 vs. without improvement: mean ferritin (95% CI): 124.2 (100.1-148.3) n=46, p=0.020]. In contrast, there was no difference in the severity of fatigue (FSS and FSMC scores) or the number of symptoms in patients with or without elevated ferritin at the first consultation (Table 4). The ANA level remained unchanged in the majority of patients (34/50, 68.0%).

**Table 4:** Differences in laboratory analysis between the first and second consultations PCS: post-COVID-19 syndrome; ANA: antinuclear antibodies; delta: value at first consultation minus the value at follow-up

Differences in laboratory analysis	PCS patients
ANA same concentration at first and second consultation, n (%)	34/50 (68.0)
ANA less concentrated at follow-up than at first consultation, n (%)	10/50 (20.0)
ANA more concentrated at follow-up than at first consultation, n (%)	6/50 (12.0)
Delta ferritin, mean (95% CI), n	88.7 (48.4-128.9), 32
Ferritin decreased, n (%)	26/32 (81.3)
Ferritin increased, n (%)	4/32 (12.5)
Ferritin unchanged, n (%)	2/32 (6.3)

## Discussion

This study focused on the course of symptoms and selected laboratory findings in PCS. The main findings are as follows: The prevalence and number of individuals with PCS symptoms decrease during the course of the disease. During a mean follow-up interval of 14 weeks, the severity of fatigue and daytime sleepiness remained stable in the majority of patients but decreased in a relatively small number of patients, whereas the severity of depression became less severe in most patients during that period. The frequency of elevated ferritin and ANA values in PCS patients is markedly increased, even months after COVID-19 infection. Our study confirms that the most commonly reported PCS symptoms are fatigue, pain, and sleep disturbances. The order of those symptoms is variable among the different studies [3,4]. However, fatigue, psychiatric (especially depressive) symptoms, respiratory symptoms (dyspnea and cough), sleep disturbances, and pain are the most common symptoms reported in almost every study, which is well in line with our findings [3,4].

The prevalence and number of symptoms in our cohort gradually decreased over time, yet more than 85% of patients reported persistent symptoms at follow-up. Consistent with other longitudinal studies, our data show that while the total number of symptoms tends to decrease over time, a substantial proportion of patients continue to experience PCS symptoms even months after the acute phase of SARS-CoV-2 infection. Fatigue, pain, and sleep disturbances remain the most frequently reported symptoms, which aligns with findings from previous studies. This highlights the long-term nature of PCS and its profound impact on patients' quality of life.

These findings are supported by a growing body of literature. For example, a German study from Heidelberg that included 91 primarily nonhospitalized patients (67.7%) reported that only 22.9% of patients were completely symptom-free after 12 months. The most common symptoms included decreased physical performance (56.3%), fatigue (53.1%), shortness of breath (37.5%), problems with concentration (39.6%), word-finding difficulties (32.3%), and sleep disturbances (26.0%) [10]. Similarly, in a study by Davis et al., 50.5% of participants reported neuropsychiatric symptoms seven months post-infection [3]. Another Italian study focusing on 121 hospitalized PCS patients reported that 82% reported persistent symptoms one year after hospitalization, with 45% still experiencing symptoms at 18 months and approximately 10% at 20 months [1]. A separate Italian cohort of nonhospitalized patients revealed that 40.5% had persistent symptoms one year after infection [24].

Notably, data are now emerging on the long-term trajectory of the PCS over two years. A European study from the Faroe Islands, which included 170 predominantly nonhospitalized patients (97.6%), reported that 38% reported persistent symptoms nearly two years (23 months) after infection, with up to 22% specifically reporting fatigue [11]. Similarly, a Spanish study by Fernández-de-Las-Peñas et al., which included 668 adult patients (67.5% nonhospitalized), reported that 63.3% reported at least one symptom two years after recovery, with fatigue (47.7%) being the most prevalent symptom among nonhospitalized patients [12]. A U.S.-based prospective cohort study by Millet et al. involving 173 adults (91 hospitalized) revealed that 23% reported at least one persistent symptom two years post-infection, with dyspnea being the most common symptom, followed by fatigue, difficulty concentrating/brain fog, memory loss, and anxiety [13].

In summary, in the current literature, on average, more than 70% of PCS patients report persistent symptoms one year after infection. This proportion decreases over time, with 10-63% of patients still experiencing symptoms after two years, depending on the study. Across studies, fatigue and neuropsychiatric symptoms, such as cognitive impairments, remain the most frequently reported complaints. These findings align closely with our observations, underscoring the chronic nature of PCS and its multifaceted impact on patients' lives. As additional key findings of our study, we observed an increase in inflammatory parameters such as ferritin and ANA.

In our cohort, elevated ferritin levels were a prominent finding, reflecting persistent inflammation in patients with PCS. A decrease in ferritin levels showed a possible association with a modest improvement in fatigue severity over time, yet fatigue remained a leading complaint in the majority of patients. However, this association was only partially statistically significant and should be interpreted with caution. While no direct correlation was found between the degree of ferritin reduction and symptom improvement, patients who reported a meaningful decrease in fatigue tended to have higher baseline ferritin levels than those without improvement. This may suggest that elevated ferritin identifies a subgroup of patients in whom fatigue is at least partially driven by inflammatory processes. The role of ferritin as an acute-phase protein that is elevated during inflammation aligns with these observations. Ferritin levels are closely associated with cytokine dysregulation, particularly involving IL-1β and IL-6, which stimulate hepcidin synthesis. Furthermore, recent studies highlight SARS-CoV-2’s intrinsic hepcidin-mimetic effect as a driver of hyperferritinemia, exacerbating chronic inflammation and contributing to PCS symptoms such as fatigue, neurocognitive impairments, and sleep disturbances [25].

The persistent elevation of ferritin levels in a significant proportion of patients with PCS has been well documented in the literature. In a German study involving a PCS and/or chronic fatigue syndrome (CFS) cohort, a positive correlation was observed between hand grip strength parameters and ferritin, suggesting a relationship between inflammation and physical performance [26]. Similarly, a Japanese study reported a positive correlation between ferritin levels during hospitalization and the presence of brain fog after COVID-19 [27]. Further evidence comes from a cross-sectional metabolic profile study, which revealed that patients with up to 90 days of PCS had significantly higher ferritin levels than did those with symptoms lasting longer than 90 days. Interestingly, patients with fewer symptoms (up to six) presented higher ferritin levels than did those with more than six symptoms [28].

These findings are broadly in line with our observations and support the hypothesis that a reduction in inflammatory activity may contribute to symptom improvement in a subset of patients. Nonetheless, the lack of consistent correlation with symptom severity and the observational design of our study limit causal interpretation. Further prospective studies with appropriate control groups are needed to clarify the role of ferritin as a biomarker for inflammatory activity and clinical recovery in PCS. However, contrasting evidence exists. A Japanese study revealed significantly higher serum ferritin levels in patients with CFS-like symptoms persisting beyond six months than in those without fatigue. This disparity underscores the complexity of the role of ferritin in the PCS and highlights the need for further research to establish its significance as a biomarker or outcome parameter [29].

Autoimmunity is another critical component of PCS pathophysiology. Elevated levels of ANA were observed in our study cohort, which is consistent with other findings in PCS patients [8,30]. Our findings align with a study that demonstrated persistently elevated ANAs for up to 12 months. In contrast to other studies that identified a correlation between ANA levels and symptoms such as fatigue, dyspnoea, and cough, we could not establish a similar association in our cohort. While ANA levels remained elevated at follow-up, there was no correlation between the severity of fatigue and ANA elevation in our patient group​​ [8,30]. These results suggest stable autoimmune activation in a significant subset of PCS patients. Similarly, more than half of our cohort exhibited elevated anti-cardiolipin/anti-glycoprotein antibodies, despite not meeting the Sapporo criteria for antiphospholipid syndrome. These observations support findings from a French study, which reported persistent IgG anticardiolipin autoantibodies in PCS patients, linking them to neurological symptoms and chronic inflammation​​ [9].

The persistence of autoantibodies and their association with ongoing inflammation highlight their potential as biomarkers for PCS and underscore the importance of further research to explore therapeutic interventions targeting autoimmune mechanisms. However, the observational nature of our study and the absence of a control group limit the ability to draw causal conclusions. Moreover, in our cohort, the presence of autoantibodies was not consistently associated with symptom severity, which weakens the argument for their role as clinically meaningful biomarkers. The stability of ANA levels over time in most patients, despite the evolution of clinical symptoms, suggests that complex and chronic immune dysregulation may contribute to PCS pathophysiology. Future studies should investigate whether these autoantibodies are precursors to the development of full-blown autoimmune diseases or represent a distinct immunopathological feature of PCS.

Our study has several limitations. Firstly, most of our patients were referred to our clinic due to fatigue and other neurological symptoms, which may have led to an overrepresentation of fatigue symptoms due to selection bias. However, it is worth noting that similar prevalence rates of fatigue have also been reported in other studies [3,4]. Second, the sample size is relatively small; however, great care was taken to ensure complete data sets (i.e., fully completed symptom scores), enabling at least a quantitative assessment of symptom severity. Third, laboratory analyses at follow-up were only performed in patients with abnormal baseline values, which may introduce selection bias and limit the reproducibility and generalizability of the laboratory findings across different cohorts and clinical settings. Furthermore, the follow-up time varied among patients and was limited to one year, which is likely too short given the often prolonged course of post-COVID-19 syndrome. Future studies with longer follow-up periods are required to better understand the long-term trajectory and underlying mechanisms of this condition, as well as the potential influence of factors such as vaccination status and viral variant on recovery.

## Conclusions

This study demonstrates that post-COVID-19 syndrome (PCS) is a long-lasting condition with substantial clinical impact, particularly in terms of fatigue, cognitive impairment, sleep disturbances, and depressive symptoms. While some improvement was seen over time, most patients in our cohort continued to experience multiple symptoms that interfered with daily functioning up to a year after the initial infection. The analysis also revealed persistent immune activation in a significant portion of patients, as reflected by elevated ferritin and autoantibody levels. These findings suggest that PCS involves complex and sustained pathophysiological processes, highlighting the need for individualized long-term care strategies and further research to better understand its mechanisms and guide targeted treatment approaches.

## Appendices

**Table 5:** Fatigue Scale for Motor and Cognitive Functions (FSMC) questionnaire All rights reserved to the original authors. The use of this instrument was licensed and authorized by the institution

Statement	Not at all true	Not really true	Somewhat true	Mostly true	Completely true
When I concentrate for a long time, I become more exhausted than others my age	☐	☐	☐	☐	☐
During periods of exhaustion, my movements become noticeably less skillful and coordinated	☐	☐	☐	☐	☐
Today, because of exhaustion, I need to take more frequent and/or longer breaks during physical activity	☐	☐	☐	☐	☐
During periods of exhaustion, I am unable to make decisions	☐	☐	☐	☐	☐
Today, in stressful situations, I feel physically exhausted faster than before	☐	☐	☐	☐	☐
Because of exhaustion, I have significantly fewer social contacts than before	☐	☐	☐	☐	☐
Today, because of exhaustion, I find it harder to learn new things	☐	☐	☐	☐	☐
Statement	Not at all true	Not really true	Somewhat true	Mostly true	Completely true
The mental demands of my work tire me more quickly than they used to	☐	☐	☐	☐	☐
During periods of exhaustion, I mainly feel it in my muscles	☐	☐	☐	☐	☐
I have more difficulty than before in maintaining prolonged physical effort	☐	☐	☐	☐	☐
When I’m stressed, my concentration decreases significantly	☐	☐	☐	☐	☐
During exhaustion, I am less motivated than others to engage in physical activities	☐	☐	☐	☐	☐
When it’s hot, my thinking slows down noticeably	☐	☐	☐	☐	☐
During exhaustion, my movements slow down noticeably	☐	☐	☐	☐	☐
Today, because of exhaustion, I am less inclined to engage in tasks that require concentration	☐	☐	☐	☐	☐
During exhaustion, I am no longer capable of reacting quickly	☐	☐	☐	☐	☐
During exhaustion, certain words escape me	☐	☐	☐	☐	☐
During exhaustion, my attention drifts off much faster than before	☐	☐	☐	☐	☐
When it’s hot, I feel physically very weak and without energy	☐	☐	☐	☐	☐
During exhaustion, I am much more prone to forget things	☐	☐	☐	☐	☐

**Table 6:** Fatigue Severity Scale (FSS) questionnaire All rights are reserved to the original authors. Use of this instrument was licensed and authorized by the institution

Statement	1	2	3	4	5	6	7
I am less motivated when I am fatigued							
Exercise brings on my fatigue							
I am easily fatigued							
Fatigue interferes with my physical functioning							
Fatigue causes frequent problems for me							
My fatigue prevents sustained physical functioning							
Fatigue interferes with carrying out certain duties and responsibilities							
Fatigue is among my three most disabling symptoms							
Fatigue interferes with my work, family, or social life							

**Table 7:** Beck Depression Inventory II (BDI-II) questionnaire All rights are reserved to the original authors and publisher. Use of this instrument was licensed and authorized by the institution

Item	Score 0	Score 1	Score 2	Score 3
Sadness	I do not feel sad	I feel sad much of the time	I am sad all the time	I am so sad or unhappy that I can't stand it
Pessimism	I am not discouraged about my future	I feel more discouraged about my future than I used to	I do not expect things to work out for me	I feel my future is hopeless and will only get worse
Past failure	I do not feel like a failure	I have failed more than I should have	As I look back, I see a lot of failures	I feel I am a total failure as a person
Loss of pleasure	I get as much pleasure as I ever did from the things I enjoy	I don't enjoy things as much as I used to	I get very little pleasure from the things I used to enjoy	I can't get any pleasure from the things I used to enjoy
Guilty feelings	I don't feel particularly guilty	I feel guilty over many things I have done or should have done	I feel quite guilty most of the time	I feel guilty all of the time
Punishment feelings	I don't feel I am being punished	I feel I may be punished	I expect to be punished	I feel I am being punished
Self-dislike	I feel the same about myself as ever	I have lost confidence in myself	I am disappointed in myself	I dislike myself
Self-criticalness	I don't criticize or blame myself more than usual	I am more critical of myself than I used to be	I criticize myself for all of my faults	I blame myself for everything bad that happens
Suicidal thoughts or wishes	I don't have any thoughts of killing myself	I have thoughts of killing myself, but I would not carry them out	I would like to kill myself	I would kill myself if I had the chance
Crying	I don't cry anymore than I used to	I cry more than I used to	I cry over every little thing	I feel like crying, but I can't
Agitation	I am no more restless or wound up than usual	I feel more restless or wound up than usual	I am so restless or agitated, it's hard to stay still	I am so restless or agitated that I have to keep moving or doing something
Loss of interest	I have not lost interest in other people or activities	I am less interested in other people or things than before	I have lost most of my interest in other people or things	It's hard to get interested in anything.
Indecisiveness	I make decisions about as well as ever	I find it more difficult to make decisions than usual	I have much greater difficulty in making decisions than I used to	I have trouble making any decisions
Worthlessness	I do not feel I am worthless	I don't consider myself as worthwhile and useful as I used to	I feel more worthless as compared to others	I feel utterly worthless
Loss of energy	I have as much energy as ever	I have less energy than I used to have	I don't have enough energy to do very much	I don't have enough energy to do anything
Changes in sleeping pattern	I have not experienced any change in my sleeping	I sleep somewhat more than usual./I sleep somewhat less than usual	I sleep a lot more than usual./I sleep a lot less than usual	I sleep most of the day./I wake up 1–2 hours early and can't get back to sleep
Irritability	I am not more irritable than usual	I am more irritable than usual	I am much more irritable than usual	I am irritable all the time
Changes in appetite	I have not experienced any change in my appetite	My appetite is somewhat less or greater than usual	My appetite is much less or greater than before	I have no appetite at all or crave food all the time
Concentration sifficulty	I can concentrate as well as ever	I can't concentrate as well as usual	It's hard to keep my mind on anything for very long	I find I can't concentrate on anything
Tiredness or fatigue	I am no more tired or fatigued than usual	I get more tired or fatigued more easily than usual	I am too tired or fatigued to do a lot of the things I used to do	I am too tired or fatigued to do most of the things I used to do
Loss of interest in sex	I have not noticed any recent change in my interest in sex	I am less interested in sex than I used to be	I am much less interested in sex now	I have lost interest in sex completely

**Table 8:** Epworth Sleepiness Scale (ESS) questionnaire All rights are reserved to the original author. Use of this instrument was licensed and authorized by the institution

Situation	Chance of Dozing
1. Sitting and reading	0 ☐ 1 ☐ 2 ☐ 3 ☐
2. Watching TV	0 ☐ 1 ☐ 2 ☐ 3 ☐
3. Sitting inactive in a public place (e.g., a theatre or meeting)	0 ☐ 1 ☐ 2 ☐ 3 ☐
4. As a passenger in a car for an hour without a break	0 ☐ 1 ☐ 2 ☐ 3 ☐
5. Lying down to rest in the afternoon when circumstances permit	0 ☐ 1 ☐ 2 ☐ 3 ☐
6. Sitting and talking to someone	0 ☐ 1 ☐ 2 ☐ 3 ☐
7. Sitting quietly after lunch without alcohol	0 ☐ 1 ☐ 2 ☐ 3 ☐
8. In a car, while stopped for a few minutes in traffic	0 ☐ 1 ☐ 2 ☐ 3 ☐

**Table 9:** Laboratory analysis performed at the first consultation CRP: C-reactive protein; TSH: thyroid-stimulating hormone; ANA: antinuclear antibodies; ANCA: antineutrophil cytoplasmic antibodies

Laboratory analysis	Reason for the analysis
Blood count	Exclusion of other pathology, especially aggravating factors for fatigue (e.g., anemia)
Kidney and liver values	Exclusion of other pathology
Electrolytes	Exclusion of other pathology
CRP	Exclusion of acute infections
TSH	Exclusion of aggravating factors for fatigue, such as hypothyroidism
Iron status (serum iron, ferritin, transferrin, transferrin saturation)	Exclusion of aggravating factors for fatigue, such as iron deficiency
Vitamin B12	Exclusion of aggravating factors for fatigue and neurological symptoms
Immunoglobulin G	Exclusion of immunodeficiency syndrome
Rheumatological screening (ANA, ANCA, anti-cardiolipin-antibodies, anti-beta-2-glycoprotein-antibodies)	Exclusion of other pathologies, such as rheumatologically disorders
